# Long-Term Effects of a Classic Ketogenic Diet on Ghrelin and Leptin Concentration: A 12-Month Prospective Study in a Cohort of Italian Children and Adults with GLUT1-Deficiency Syndrome and Drug Resistant Epilepsy

**DOI:** 10.3390/nu11081716

**Published:** 2019-07-25

**Authors:** Ramona De Amicis, Alessandro Leone, Chiara Lessa, Andrea Foppiani, Simone Ravella, Stefano Ravasenghi, Claudia Trentani, Cinzia Ferraris, Pierangelo Veggiotti, Valentina De Giorgis, Anna Tagliabue, Alberto Battezzati, Simona Bertoli

**Affiliations:** 1 International Center for the Assessment of Nutritional Status (ICANS), Department of Food Environmental and Nutritional Sciences (DeFENS), University of Milan, Via Sandro Botticelli 21, 20133 Milan, Italy; 2 Human Nutrition and Eating Disorder Research Center, Department of Public Health, Experimental and Forensic Medicine, University of Pavia, Via Agostino Bassi 21, 27100 Pavia, Italy; 3 Pediatric Neurology Unit, Vittore Buzzi Hospital, Via Lodovico Castelvetro 32, 20154 Milan, Italy; 4 Biomedical and Clinical Sciences Department, Luigi Sacco Hospital, University of Milan, via G. B. Grassi 74, 20157 Milan, Italy; 5 Department of Child Neurology and Psychiatry, IRCCS Mondino Foundation, Via Mondino 2, 27100 Pavia, Italy

**Keywords:** drug-resistant epilepsy, GLUT1-Deficiency Syndrome, ketogenic diet, leptin, ghrelin

## Abstract

The classical ketogenic diet (cKD) is an isocaloric, high fat, very low-carbohydrate diet that induces ketosis, strongly influencing leptin and ghrelin regulation. However, not enough is known about the impact of a long-term cKD. This study evaluated the effects of a 12-month cKD on ghrelin and leptin concentrations in children, adolescents and adults affected by the GLUT1-Deficiency Syndrome or drug resistant epilepsy (DRE). We also investigated the relationship between the nutritional status, body composition and ghrelin and leptin variations. We carried out a longitudinal study on 30 patients: Twenty-five children and adolescents (15 females, 8 ± 4 years), and five adults (two females, 34 ± 16 years). After 12-monoths cKD, there were no significant changes in ghrelin and leptin, or in the nutritional status, body fat, glucose and lipid profiles. However, a slight height z-score reduction (from −0.603 ± 1.178 to −0.953 ± 1.354, *p* ≤ 0.001) and a drop in fasting insulin occurred. We found no correlations between ghrelin changes and nutritional status and body composition, whereas leptin changes correlated positively with variations in the weight z-score and body fat (*ρ =* 0.4534, *p =* 0.0341; *ρ =* 0.5901, *p =* 0.0135; respectively). These results suggest that a long-term cKD does not change ghrelin and leptin concentrations independently of age and neurological condition.

## 1. Introduction

The classic ketogenic diet (cKD) is an isocaloric, high-fat, very low-carbohydrate and normal-protein diet. It requires all foods and beverages to be carefully calculated and precisely weighed on a gram scale in order to obtain a specific ratio between fats (gr) and carbohydrates (gr) plus proteins (gr), generally equal to 3:1 or 4:1 [1]. It has been used safely and effectively for decades as a recognized treatment for drug-resistant epilepsy (DRE) [2,3,4], in GLUT1-Deficiency Syndrome (GLUT1-DS) [5,6] and in pyruvate dehydrogenase complex deficiency (PDCD) [7,8]. While in DRE it remains currently unclear how the cKD works despite a much better understanding of anticonvulsant mechanisms [4], in the GLUT1-DS and PDCD it is the treatment of choice in order to switch brain metabolism from glucose to ketone bodies (KBs) and leading to a powerful improvement in neurologic symptoms. In fact, GLUT1-DS is a rare disease caused by mutations in the SLC2A1 gene that encodes the glucose carrier protein type 1 (GLUT1), the main carrier of glucose across the blood-brain barrier, which is characterized by early-onset seizures, developmental delay, and a complex movement disorder [9]. cKD has also been explored in other neurological and neurodegenerative diseases, such as Alzheimer’s, Parkinson’s, amyotrophic lateral sclerosis, spinal cord injury, glycogenosis [10,11], and more recently in metabolic syndrome and type 2 diabetes [12,13].

The cKD produces the highest ketogenic effect among the various ketogenic diets (KDs). It induces a constant production of KBs, called ketosis, aimed at mimicking the starvation state while providing adequate calories to support growth and energy needs in childhood and adult age, respectively [2,14]. 

KBs, such as acetone, acetoacetate and β-hydroxybutyrate, are involved in several mechanisms [15]. Firstly, they have a “neuroketotherapeutic” effect [16], modulating the release of inhibitory neurotransmitters, reducing neuronal excitation and seizure activity. They also have an antioxidant effect, reducing the production of reactive oxygen species (ROS) and damage caused by them, and increasing mitochondrial biogenesis and function [16]. In the GLUT1-DS and pyruvate dehydrogenase complex deficiency, KBs are the main neuronal energy source [17]. Finally, KBs also appear to decrease the pro-inflammatory cytokines that have an anti-inflammatory effect [15].

cKD may possibly also influence the modulation of multifunctional hormones such as ghrelin and leptin [18,19,20,21]. These hormones are involved in food intake, glucose metabolism, neuronal activity and also the maintenance of nutritional status [22,23]. Specifically, KBs may be implicated in the reduction of food intake and consequent weight loss in adults [24] or in the potential adverse effect of growth retardation in children during a long-term cKD [25,26,27].

Ghrelin is the only gastrointestinal peripheral peptide with orexigenic properties, whose circulating plasma levels are increased by fasting and decreased by feeding [22]. Ghrelin’s concentration is also inversely correlated with body weight and age [22,28]. It has been found to be an endogenous ligand for the growth hormone secretagogue receptor (GHSR 1a), which can stimulate the release of growth hormones (GH) from the anterior pituitary gland [29], inducing a broad spectrum of functions in relation to food intake, adiposity and glucose metabolism [22]. In addition, it has anti-seizure effects, which stimulate the release of protective neurotransmitters, which help to improve the survival and proliferation of neurons [18,30]. Previous studies have investigated the short-term changes of ghrelin during the cKD with contrasting results [18]. In fact, in children with DRE, decreased levels of ghrelin were found after 3-months of cKD [19], whereas in adults the level increased [18,31].

Leptin, on the other hand, is an anorexigenic hormone, which is produced in small amounts at a central level and widely released by a white adipose tissue into the circulatory system in order to suppress hunger and, consequently, food intake [28]. Its blood concentration is directly proportional to the amount of body fat mass, which is why its plasma levels increase in obese subjects [28]. The most significant roles of leptin include the regulation of energy homeostasis, the neuroendocrine function and metabolism [32], as it interacts with other hormonal mediators and regulators of energy status and metabolism such as insulin, glucagon, insulin-like growth factors and growth hormones [23]. Some studies have hypothesized an increase in leptin in humans on the cKD, as shown also in rodents placed on a cKD [33,34]. However, most human studies have shown that leptin decreases during KD, probably due to the concomitant reduction of the adipose tissue [18,20,35].

Although ghrelin and leptin changes during cKD are of great interest due to their impact on the nutritional and metabolic outcomes of cKD and their potential effects as therapeutic targets in neurological diseases [30], to the best of our knowledge they have been analysed several times in DRE and GLUT1-DS [19,20,21], where the cKD must be followed for long term, but it is difficult to draw substantial conclusions due to short follow-up examinations or incomplete data.

We therefore carried out a longitudinal study to investigate the effects of a 12-months cKD on ghrelin and leptin concentrations in children, adolescents and adult patients affected by GLUT1-DS or DRE. We also explored the relationship between the nutritional status, body composition and ghrelin and leptin concentrations at the baseline as well as at 12 months.

## 2. Materials and Methods 

### 2.1. Study Design

This was a 12-month, prospective, multi-centre study in patients with GLUT1-DS and DRE treated with isocaloric cKD, according to the following criteria:

- age > five months;
- absence of absolute contraindications, such as carnitine deficiency, β-oxidation defects, pyruvate carboxylase deficiency, and porphyria, as stated in the latest consensus [8];
- parents’ or caregivers’ compliance ensured by the physician who well explained to caregivers their critical role in the administration of cKD to their children or parents, including the involvement of time in the preparation of meals for the child who will require meals other than the rest of the family, the cost of food, the avoidance of carbohydrates, additional supplementation and potential side effects [8].

The main outcome measures were the changes in ghrelin and leptin from the baseline. The assessment included neurological examinations; ghrelin and leptin dosage, glucose, insulin, and lipid profile (triglycerides [TGs], total cholesterol [TC], low-density lipoprotein cholesterol [LDL-C], and high-density lipoprotein cholesterol [HDL-C]); nutritional status evaluation by anthropometry and abdominal body fat distribution by ultrasonography. In children and adolescents, we conducted the assessment at the baseline and six and 12 months after cKD intervention, while in adults the assessment was conducted at the baseline and after 12 months.

The study was approved by the ethical committee of the Fondazione IRCCS Policlinico San Matteo of Pavia (reference number 20180083746) and complied with all tenets of the Helsinki declaration. All the children’s and adolescents’ caregivers and adult patients provided written informed consent before the beginning of the study.

### 2.2. Settings

Patients were recruited at the Department of Child Neurology and Psychiatry, Fondazione IRCCS Istituto Neurologico C.Mondino in Pavia, and at the Pediatric Neurology Unit, “V. Buzzi” Hospital in Milan, Italy from October 2010 to February 2018.

KD treatment was implemented at the Human Nutrition and Eating Disorders Research Centre in Pavia, Italy.

Biochemical and anthropometric measurements were performed at the International Center for the Assessment of Nutritional Status (ICANS) of the University of Milan, Italy. 

### 2.3. Patients

We prospectively enrolled 30 patients: Twenty-five children and adolescents (15 females and 10 males, mean age 8 ± 4 years), of which 19 were affected by GLUT1-DS, and five adults (two females and three males, mean age 34 ± 16 years), were all diagnosed with GLUT1-DS. 

All the patients met the clinical criteria for the DRE and GLUT1-DS diagnosis:

- concerning GLUT1DS, all patients underwent a lumbar puncture in the fasting state (after 5–6 h of fasting); a blood sample for glucose measurement was obtained immediately before the procedure to avoid stress-related hyperglycaemia [9]. A cerebrospinal fluid-to-blood glucose rate of <0.6 was considered suspicious for GLUT1 DS. Subsequently, for a definitive confirmation, all the patients were submitted to the SLC2 A1 mutation analysis;
- DRE was defined as “failure of adequate trials of two tolerated and appropriately chosen and used antiepileptic drugs schedules to achieve sustained seizure freedom” according to the Current International League Against Epilepsy (ILAE) Consensus [36]. It was diagnosed with the Lennox Gastaut Syndrome (LGS), Electrical Status Epilepticus during Sleep (ESES), and epileptic encephalopathy. All patients underwent magnetic resonance imaging (MRI) in order to classify the cortical malformation. All patients were treated with specific pharmacotherapy. Seizure types and epilepsy syndromes were classified according to the criteria proposed by the International League Against Epilepsy (ILAE) [36].

### 2.4. Ketogenic Diet

A non-fasting dietary protocol with an at-home gradual increase of the ketogenic ratio was implemented at the Human Nutrition Research Centre outpatient clinic according to a standardized protocol [37].

At the baseline, we evaluated the usual caloric intake and food intolerances and preferences for each patient using seven-day weighted food diaries analysed by a dietitian using the WinFood version 3.0. The initial calorie prescription was based on age-related energy requirements considering weight and height (both current and recent trends), and physical activity levels. The macronutrient composition included a minimum of 0.8 g–1 g of protein from animal sources per kilogram of body weight (e.g., eggs, milk, meat, poultry and fish). 

All patients and caregivers received pre-diet counselling in order to ensure understanding regarding the meal preparation, cooking strategy, importance of avoiding carbohydrates, additional supplementation, and potential side effects. 

All patients started a 1:1 cKD at home and gradually proceed to 2:1, 3:1 or 4:1 ketogenic ratio in order to obtain blood values of beta-hydroxybutyrate >2.0 mml/L. All children’s and adolescents’ caregivers and adults were instructed to check capillary ketonemia and ketonuria on a daily basis during the first month and then twice a week, and to report the values in a specific format. To measure the dispersion of the ketonemia during cKD, for each patient we calculated the coefficient of variation (CV) at the baseline and at 12 months as the ratio of the standard deviation to the mean of the reported KBs. Ketonemia at the baseline was considered as the average of the daily KBs of the first two weeks after the induction phase, while the final as the average of the last two weeks before the evaluation at 12 months. 

24-h dietary recalls were collected during each follow-up examination to evaluate the compliance.

### 2.5. Main Outcome Measurements

#### 2.5.1. Neurological Assessment

In accordance with the 2011 Italian consensus on KD therapy [38], neurologic evaluations and electroencephalography (EEG) were performed after one, six and twelve months. The following neurological symptoms were monitored: Paroxysmal dyskinesia, dysarthria, ataxia, spasticity dystonia, muscle strength, as well as seizure types and frequency. Children’s and adolescents’ caregivers and adult patients completed a daily record regarding alertness, activity and seizure occurrence.

#### 2.5.2. Anthropometric Measurements

Anthropometric measurements were taken by the same trained dietician in accordance with conventional criteria and measuring procedures [39].

Body weight (BW, kg) and body height (BH, cm) were measured to the nearest 100 g and 0.5 cm, respectively. The body mass index (BMI) was calculated using the formula: BW (kg)/BH^2^ (m^2^). For children and adolescents, sex-specific BMI-for-age Z scores were calculated from the growth charts of 2000 Centers for Disease Control and Prevention (CDC) [40]. In accordance with CDC guidelines, a z-score of ≤−2 was considered as severely underweight, a score between −2 and −1 was considered underweight, between −1 and +1 was considered as normal weight, +1 and +2 was considered overweight, and >+2 was considered as obese. For adults, the BMI was classified into four categories: Underweight (BMI <18.5 kg/m^2^), normal weight (BMI 18.5 kg/m^2^–24.9 kg/m^2^), overweight (BMI 25.0 kg/m^2^–29.9 kg/m^2^), and obese (BMI >30.0 kg/m^2^) [41].

Skinfold thickness was measured as proposed in a previous work [36] by a Holtain LTD caliper, at the triceps, biceps, subscapular, and supra-iliac sites on the non-dominant side of the body. All measurements were taken in triplicates for all sites, and the average of the three values was calculated. The intra-observer variation for the skinfold measurement ranged between 2.5% and 2.9%. The body density and fat mass (FM, kg) were calculated for children and for adolescents and adults by the Brook method [42] and the Brozek Formula [43], and by the Durnin and Womersley method [44] and the Siri formula [45], respectively. We calculated the fat mass index (FMI, kg/m^2^) in children and adolescents by dividing FM by the squared height.

#### 2.5.3. Abdominal Fat Distribution

Abdominal subcutaneous fat (SAT) and abdominal visceral fat (VAT) were measured on fasting patients by the same operator using a Logiq 3 Pro Ultrasonography equipped with a 3.5 MHz convex-array probe and with a 7.5 MHz linear probe (GE Healthcare, Milwaukee, WI, USA) following a validated standardized protocol [46]. Specifically, SAT was measured as the distance between the epidermis and the external face of the rectus abdominis muscle. VAT was measured as the distance between the anterior wall of the aorta and the posterior surface of the rectus abdominis muscle measured at the level of the xipho-umbilical line or linea alba. The within-day intra-operator coefficient of variation for repeated measures of VAT and SAT in our laboratory is 0.8%.

#### 2.5.4. Biochemical Parameters

Fasting blood samples were taken by venepuncture of the antecubital vein in either the sitting or lying position, using vacuum tubes. After centrifugation (800× *g* 10 min at 5 °C), aliquots of samples were stored at 80 °C until further analysis.

Ghrelin and leptin (pg/mL) were measured using an enzymatic immunoassay kit (R & D Systems; Wiesbaden, Germany). Interassay precision (CV) was 3.3%, and 3.4%, respectively. 

We used an autoanalyzer (Cobas Integra 400 plus, Roche Diagnostics, Mannheim) to determine the serum glucose, TC, HDL-C, LDL-C, TG concentrations. Circulating insulin was measured in duplicate by an autoanalyzer (Cobas e411 Hitachi, Roche Diagnostics). The homeostatic model assessment-insulin resistance (HOMA-IR) was calculated as [fasting glucose (mg/dL) × fasting insulin (mU/L)/405] [47].

High glucose was defined as glucose ≥100 mg/dL [48,49], high insulin as >23 uU/mL, and high HOMA-IR as ≥3.16 for children or ≥2.5 for adults [47,50]. High TC was defined as ≥200 mg/dL, high LDL-C as > 130 mg/dL, and high TG as >150, while low HDL as < 40 mg/dl for male and <50 mg/dL for females [48,49].

Capillary ketonemia was measured with an in vitro diagnostic medical device for β-ketone self-testing (GlucoMen LX PLUS, Menarini Diagnostics, test range 0.1 mmol/L–8.0 mmol/L). Ketonuria was measured by a urine ketone stick test (Ketostix^®^, Bayer Diabetes, Berkshire, UK).”

### 2.6. Statistical Analysis 

Continuous variables are presented as a mean ± standard deviation. Leptin, insulin and HOMA-IR were not normally distributed and were normalized using log-transformation. Similarly, ghrelin was normalized using a square root transformation. An independent T-test was used to compare the means of nutritional and biochemical variables between GLUT1-DS and DRE children. A one-way repeated measures ANOVA with Bonferroni’s test for comparison was run to determine if there were differences in the variables of interest during the 12-month ketogenic dietary treatment. The Spearman correlation was used to investigate the association of ghrelin and leptin concentrations and changes in the nutritional status and body composition. All P-values were two-tailed, and *p* < 0.05 was considered significant. Statistical analysis was performed using Stata 12.1 (Stata Corporation, College Station, TX, USA).

## 3. Results

### 3.1. Pre-Intervention

Table 1 shows the characteristics of the patients at recruitment. 

Among the children and adolescents, five patients were underweight (BMI z-score < −1,9) according to the CDC BMI standards, one adult was underweight (BMI < 18.5), none were overweight or obese. None of the children, adolescents or adults showed fasting glucose >100 mg/dL. Three children and one adult had a high HOMA-IR index value (>2.5); three children and one adult had both TC and LDL-C levels above the cut off; three children had low HDL-C levels, and none of them showed triglyceride levels above the cut off. Both GLUT1-DS and DRE children and adolescents reported ghrelin and leptin values in the normal range (395.19 ± 204.34 pg/mL; 6.82 ± 2.70 ng/mL; respectively) [51,52]. No differences in the nutritional status, body composition and biochemical parameters were found between GLUT1-DS and DRE children and adolescents.

### 3.2. Intervention

Table 2 shows the macronutrient composition and ketogenic ratio of the cKD implemented by the dietitian. 

There were no differences in the diet composition between GLUT1-DS and DRE children and adolescents. All patients completed the 12-month protocol.

### 3.3. Post-Intervention

Children, adolescents and adults reached the therapeutic range of KBs (beta-hydroxybutyrate >2.0 mmol/L) and tolerated the diet well. Table S1 shows changes of the diet composition at six and 12 months.

Neurological data on the effect of cKD have already been published in two papers by our research group [53,54].

Table 3 shows changes at six and 12 months from the beginning of the cKD.

After 6-months of cKD, KBs increased significantly both in children and adolescents and in adults (from 0.06 ± 0.05 to 2.78 ± 0.64 mmol/L, *p*-value = < 0.0001; from 0.05 ± 0.05 to 2.35 ± 1.36 mmol/L, *p*-value = 0.022, respectively), and remained stable at 12-months, in line with the diet. At the end of the induction phase, KBs were 2.99 ± 0.78 mmol/L with a CV equal to 19.3 ± 8.6%. At 12 months KBs were 2.89 ± 0.66 mmol/L with a CV equal to 20.4 ± 10.0% (*p*-value = 0.904).

Concerning children and adolescents, in the first six months, no significant differences were found in the BMI z-score and the amount of body fat and its abdominal distribution. However, we found a slight reduction of height z-score after 12 months of cKD (from −0.603 ± 1.178 to −0.953 ± 1.354, *p*-value = <0.001). Regarding the biochemical parameters, although serum glucose did not change, fasting insulin decreased after cKD, and HOMA-IR was significantly modified. Neither the lipid nor leptin and ghrelin profiles changed significantly. None of these variables changed even at 12 months.

Similarly, in adults after 12-month cKD, no significant changes were observed in the body composition and abdominal fat amount (SAT and VAT). Maintenance of serum glucose occurred, while insulin and HOMA-IR changed significantly. Neither ghrelin nor leptin and nor TC/LDL/HDL/TG changed significantly. 

### 3.4. Associations between Nutritional Status, Body Composition, Ghrelin and Leptin

Table 4 and Table 5 show the associations between the nutritional status and body composition with concentrations and changes in ghrelin and leptin pre-intervention and post-intervention, both in children and adults, respectively. 

At pre-intervention, in children and adolescents, the ghrelin concentration correlated negatively with the age and BMI z-score (*ρ =* −0.5180, *p =* 0.0080; *ρ =* −0.4493, *p =* 0.0410; respectively) and as expected, the leptin concentration correlated positively with the weight z-score, BMI z-score and body fat amount (*ρ =* 0.4707, *p =* 0.0234; *ρ =* 0.6351, *p =* 0.0020; *ρ =* −0.5896, *p =* 0.0101; respectively). Insulin correlated negatively with ghrelin concentration (*ρ =* 0.5134, *p =* 0.0210).

At post-intervention, no correlations were found between Δghrelin concentration and changes in the nutritional status and body composition variables, whereas the Δleptin concentration correlated positively with variations in the weight z-score and body fat (*ρ =* 0.4534, *p =* 0.0341; *ρ =* 0.5901, *p =* 0.0135; respectively). 

In adults, no correlations were found between the nutritional status, body composition, leptin, and ghrelin either in pre-intervention or in post intervention.

## 4. Discussion

The main aim of our longitudinal study was to investigate the effects of a 12-month cKD on ghrelin and leptin concentrations in children, adolescents and adult patients affected by GLUT1-DS or DRE. To the best of our knowledge, this is the first that it has been demonstrated that in the long-term, cKD did not change ghrelin and leptin concentrations independently of age and the neurological condition for which the cKD was prescribed.

These results are in contrast with those obtained by Marchiò et al. [19]. These authors studied only children affected by DRE and found a significant reduction in both ghrelin and des-acyl ghrelin plasma levels during a cKD. However, their study was short-term with a follow-up of only three months, and the differences in ghrelin concentrations are probably due to an initial metabolic adaptation to the high fat content of the cKD. Other authors, such as Sumithran [55] and Nymo et al. [56], examined changes in all appetite-regulating peripheral hormones and found a significant increase in ghrelin associated with a high change in body composition after a KD. However, they examined the effects of a very low energy diet (VLED) on overweight and obese adults with an only 8–10-week follow-up. VLEDs are also called “starvation diets” due to their extremely low daily amount of energy, equal to about 800 kcal/day, one third of the average energy requirement for a man (2500 kcal) and half for a woman (2000 kcal). VLEDs are usually undertaken by overweight and obese patients for rapid weight loss and, due to the strong reduction in food calories that VLED requires, cannot be followed for long periods. Compared to cKD, VLEDs provide a higher protein content (1.5 g/kg/day), in order to accelerate fat oxidation and KB production and better control hunger and satiety. 

Regarding leptin, these results confirm our previous ones in a smaller sample (*n* = 10) and with a shorter follow-up (3-months) [34] but are in contrast with the results of Lambrechts et al. [20]. These authors found a leptin decrease after a 12-month KD in patients affected by DRE aged between one and 40 years, following various KDs, such as cKD and medium chain triglyceride (MCT) KD which uses a fat supplement consisting only of MCT fats that produce ketones more easily than the long-chain ones used in the cKD. However, their KD was different from the cKD, because they gradually added five-gram steps to the diet with a maximum of 20 g/day of carbohydrates when adequate ketosis was not reached or to prevent weight loss. Rauchenzauner et al. [21] also reported decreased leptin levels in children affected by GLUT-1 DS and treated with a cKD for at least six months: this change was not dependent on weight loss.

The different types of KDs examined, the various study-designs and the different ages and diseases of the examined patients, could explain these contrasting results. 

Our study showed stable ghrelin and leptin concentrations, probably due to the isocaloric cKD, which permitted a stable KB production without impairing the nutritional status. Indeed, the BMI did not change significantly, and the amount and distribution of body fat also remained stable, corroborating our previously published data on the short-term effects of cKD on the nutritional status and body composition in children affected by GLUT1-DS [28]. We found, as expected [23,32], that the leptin concentration changes were positively associated with the weight gain and the increase in body fat amount (*ρ* = −0.4534, *p* = 0.0344; *ρ* = 0.5901, *p* = 0.0135; respectively). Some authors have hypothesized a downregulated concentration in ghrelin and, in contrast, an increase in leptin levels during KDs ad libitum due to the increase in dietary fat intake and, consequently, a higher body fat mass [18,33]. However, the strict and constant evaluation of both the caloric and fatty acid intakes, probably led to the maintenance of the nutritional status, lipid profile and both ghrelin and leptin levels in the 12-month cKD. These data, taken together, suggest that, weight loss, probably including both fat mass and fat free mass loss, as occurred during the VLED, rather than KBs, affects the ghrelin and leptin concentration, acting on hungry and satiety control. During a normocaloric cKD, KBs do not seem to directly regulate body weight and body composition.

After 12 months of cKD, in our sample of children and adolescents, we found a statistically significant reduction in the height z-score, with a magnitude of variation of very low clinical impact (−0.19 ± 0.15 height z-score). A decline in height [27] or weight and height [57,58,59,60,61] with the KD has been previously described in epileptic patients on the diet for more than six months; and the percentage of affected patients varies between 6% and 30% in recent long-term studies [62,63,64]. 

Data on factors that may affect the growth of children treated with the KD (e.g., ketosis or nutrient adequacy) remain scarce and contrasting [26,65]. However, a reduction in both ghrelin and insulin, both of which are hormones stimulating GH concentration, has been hypothesized [29,66]. Our study does not suggest the potential role of ghrelin on the delay of growth, because we did not find either a significant change in ghrelin over the 12 months (*p* = 0.693), or an association with the ghrelin and height z-score changes (*ρ* = 0.0019, *p* = 0.9963). As for insulin, although we found a significant difference between the baseline and 12 months (*p* < 0.001), insulin was not significantly associated with the height z-score changes (*ρ* = −0.1182, *p* = 0.6751). However, we cannot rule out that insulin changes may have had an effect during cKD and led to growth failure. This is because from a metabolic point of view not only is the fasting concentration of insulin relevant but also the after-meal concentration. The chronic very low amount of carbohydrates of the cKD could significantly impair insulin secretion after a meal. Further studies on the effects of the ketogenic diet on insulin secretion are needed to clarify this issue. 

Regarding the biochemical parameters, children, adolescents and adults reported unchanged fasting glucose, while there was a drop in the fasting insulin and a relative improvement in HOMA-IR, as previously demonstrated [34]. The three children and one adult (13%) with high TC and LDL at the baseline, reported a rise (+25%) in both levels, while triglycerides remained in the normal range. In the other patients, the lipid profile did not change, despite the daily high-fat dietary consumption, in contrast with a previous study [67] that found a substantial increase in TC, HDL, LDL and TG at six months. Our results agree with a previous study that found no significant changes in TC or LDL-C in 10 prepubertal children affected by GLUT1-DS after 10 years of a cKD and did not identify any cardiovascular risks [6].

One of the strengths of the study is the quality of data collected: All the biochemical measurements and dosages were collected in the same centre, thus guaranteeing less variability. It is also the first time that ghrelin, leptin, insulin and nutritional status parameters have been measured all together and both in DRE and, above all, in GLUT1-DS. Finally, our study involved what we believe to be the longest follow-up ever reported in the literature. However, a number of potential limitations need to be addressed. A control group was not included in the study, since this is difficult to achieve especially because the GLUT1-DS management guidelines require the use of KD from the first day of diagnosis [5]. The sample size is also globally small, especially in adults, and thus more studies are needed. 

## 5. Conclusions

The present study is, to the best of our knowledge, the first to investigate the effects of a 12-month cKD on ghrelin and leptin concentrations in children, adolescents and adults affected by GLUT1-DS or DRE. We found no significant changes and associations between the ghrelin and leptin and nutritional status changes, suggesting that these appetite-regulating peripheral hormones are not downregulated by chronic ketosis or by a very high fat diet. Our results contribute to the growing literature on changes during cKD of these two peripheral hormones implicated in several metabolic outcomes, and thus to a better understanding of the long-term effects of a cKD and how it could be implemented in other diseases.

## Figures and Tables

**Table 1:** Characteristics of the patients at recruitment.

	Children and Adolescents							Adults	
	Total		GLUT1-DS		DRE			GLUT1-DS	
	(*n* = 25)		(*n* = 19)		(*n* = 6)			(*n* = 5)	
	Mean	Sd	Mean	Sd	Mean	sd	*p* Value	Mean	sd
Age (years)	8	4	8	4	6	5	0.487	34	16
Nutritional status and body composition									
Weight (kg)	27.5	20.4	28.2	19.0	25.4	24.5	0.573	63.5	9.1
Weight z-score	−0.365	1.830	−0.340	1.868	0.454	1.888	0.761	-	-
Height (cm)	120.0	29.1	122.5	27.8	111.9	34.3	0.751	168.4	6.9
Height z-score	−0.603	1.177	−0.508	1.162	−0.825	1.294	0.424	-	-
BMI (kg/m^2^)	16.5	4.3	16.5	4.3	16.4	4.9	0.745	22.6	4.9
BMI z-score	−0.587	1.839	−0.423	1.353	−1.141	2.685	0.283	-	-
Body fat (%)	20.8	7.0	21.0	6.2	34.4	11.6	0.773	27.2	12.8
FMI (kg/m^2^)	5.4	2.6	5.2	2.5	5.9	3.1	0.874	-	-
SAT (cm)	0.8	0.7	0.8	0.8	0.7	0.7	0.561	5.4	6.9
VAT (cm)	2.9	1.6	2.7	1.7	3.5	1.1	0.307	9.65	11.2
Biochemical parameters									
Serum glucose (mg/dL)	83	11	82	12	89	8	0.255	90	6
Log-Insulin (mU/L)	1.34	1.19	1.33	1.27	1.40	0.94	0.483	2.14	0.43
Log-HOMA-IR	0.24	1.25	0.27	1.35	0.64	0.11	0.473	0.63	0.45
TC (mg/dL)	170	37	174	39	154	20	0.255	201	14
HDL-C (mg/dL)	58	14	60	15	49	9	0.631	68	8
LDL-C (mg/dL)	99	33	100	36	98	16	0.269	121	12
TG (mg/dL)	61	18	60	17	66	25	0.743	57	14
√ghrelin (pg/mL)	19.74	6.44	17.98	5.91	25.3	4.930	0.895	17.84	2.83
Log-leptin (pg/mL)	8.58	1.16	8.60	1.20	8.49	1.12	0.796	9.26	1.30
KBs (mmol/L)	0.06	0.05	0.06	0.04	0.06	0.05	0.708	0.05	0.05

BMI = body mass index; FMI = fat mass index; SAT = abdominal subcutaneous fat; VAT= abdominal visceral fat; HOMA-IR = homeostatic model assessment-insulin resistance; TC = total cholesterol, LDL-C = low-density lipoprotein cholesterol; HDL-C = high-density lipoprotein cholesterol; TG = triglycerides; KBs = ketone bodies.

**Table 2:** Diet composition.

	Children and Adolescents (*n* = 25)							Adults	
	Total		GLUT1-DS		DRE			GLUT1-DS	
	(*n* = 25)		(*n* = 19)		(*n* = 6)			(*n* = 5)	
	Mean	Sd	Mean	sd	Mean	sd	*p*-Value	Mean	sd
Energy intake (kcal/day)	1373	518	1433	535	1132	401	0.315	1938	325
Energy intake/BW (kcal/kg)	61.5	21.3	59.5	17.5	69.2	34.2	0.662	31.6	9.5
Protein (g/day)	26.4	12.4	27.1	13.5	23.9	7.2	0.510	59.0	16.9
Protein/BW (g/kg)	1.2	0.5	1.1	0.3	1.5	0.9	0.348	1.0	0.3
Protein (%)	8	2	7	1	8	2	0.403	13	5
Fat (g/day)	131.3	51.0	137.4	51.8	107.0	43.6	0.316	177.6	43.5
Fat/BW (g/kg)	5.0	2.0	5.7	1.7	6.4	3.2	0.753	2.9	1.1
Fat (%)	86	5	86	3	85	8	0.697	82	8
SFA (g/day)	47.4	19.0	47.1	20.7	39.0	17.5	0.371	40.3	7.6
SFA/BW (g/kg)	2.0	0.7	1.7	0.8	1.7	0.8	0.407	0.6	0.2
SFA (%)	28	8	28	7	28	8	0.796	19	5
Carbohydrate (g/day)	21.6	12.6	22.4	11.7	18.6	17.1	0.641	20.1	8.7
Carbohydrate/BW (g/kg)	1.1	0.9	1.0	0.7	1.4	1.6	0.680	0.4	0.1
Carbohydrates (%)	7	4	7	3	7	6	0.873	4	2
Ketogenic ratio	2.9	0.8	2.9	0.7	2.8	1.1	1.000	2.5	0.9

BW = body weight (kg); SFA = saturated fatty acids.

**Table 3:** Time course of nutritional status, body composition and biochemical parameters during 12-month classical ketogenic diet (cKD).

	Children and Adolescents (*n* = 25)							Adults (*n* = 5)				
	Baseline		6 Months		12 Months			Baseline		12 Months		
	Mean	Sd	Mean	sd	Mean	sd	*p*-Value	Mean	sd	Mean	sd	*p*-Value
Nutritional status and body composition												
Weight (kg)	27.5	20.4	29.4	20.1	29.0	18.5	0.124	63.5	9.1	62.4	5.0	0.469
Weight z-score	−0.365	1.830	−0.605	1.320	−1.032	1.475	0.146	-	-	-	-	-
Height (cm)	120.0	29.1	124.0	30.8	125.2	28.4	<0.001	168.4	6.9	170.2	7.3	0.512
Height z-score	−0.603	1.177	−0.505	1.014	−0.953	1.354	<0.001	-	-	-	-	-
BMI (kg/m^2^)	16.5	4.3	17.1	3.5	16.7	3.3	0.694	22.6	4.9	21.7	3.4	0.276
BMI z-score	−0.587	1.839	−0.228	1.337	−0.609	1.295	0.917	-	-	-	-	-
Body fat (%)	20.8	7.0	21.7	6.1	21.2	5.8	0.712	27.2	12.8	23.0	10.1	0.349
FMI (kg/m^2^)	5.4	2.6	6.01	2.6	6.35	1.9	0.207	-	-	-	-	-
SAT (cm)	0.8	0.7	0.7	0.5	0.8	0.4	0.875	5.4	6.9	1.5	1.2	0.492
VAT (cm)	2.9	1.6	3.0	1.2	2.6	1.2	0.921	9.65	11.2	3.7	0.9	0.183
Biochemical parameters												
Serum glucose (mg/dL)	83	11	78	8	79	12	0.094	90	6	96	10	0.354
Log-Insulin (mU/L)	1.34	1.19	0.89	0.83	1.10	0.93	<0.001	2.14	0.43	1.66	0.37	<0.001
Log-HOMA-IR	0.24	1.25	−0.78	0.89	−0.54	1.01	<0.001	0.63	0.45	0.21	0.40	0.034
TC (mg/dL)	170	37	178	39	174	40	0.688	201	14	188	34	0.348
HDL (mg/dL)	58	14	61	16	62	19	0.207	68	8	66	11	0.135
LDL (mg/dL)	99	33	110	32	102	32	0.546	121	12	123	31	0.963
TG (mg/dL)	61	18	70	33	67	29	0.294	57	14	57	25	0.990
√ghrelin (pg/mL)	19.74	6.44	18.77	6.23	18.90	6.17	0.693	17.84	2.83	15.06	3.43	0.102
Log-leptin (pg/mL)	8.58	1.16	8.57	1.02	8.68	0.90	0.808	9.26	1.30	9.13	1.01	0.898
KBs (mmol/L)	0.06	0.05	2.78	0.64	2.45	1.77	<0.001	0.05	0.05	2.65	0.82	0.022

BMI = body mass index; SAT = abdominal subcutaneous fat; VAT = abdominal visceral fat; HOMA-IR = homeostatic model assessment-insulin resistance; TC = total cholesterol, LDL-C = low-density lipoprotein cholesterol; HDL-C = high-density lipoprotein cholesterol; TG = triglycerides; KBs = ketone bodies.

**Table 4:** Associations between nutritional status, body composition, ghrelin and leptin at the baseline and 12-months in children and adolescents.

Pre-Intervention											Post-Intervention							
		Age	Weight z-Score	Height z-Score	BMI z-Score	Body fat	√ghrelin	LOG-Leptin	Log-Insulin			ΔWeight z-Score	ΔHeight z-Score	ΔBMI z-Score	ΔBody fat	Δ√ghrelin	ΔLog-Leptin	ΔLog-Insulin
Age	ρ	1.0000																
	*p*-value																	
Weight z-score	ρ	0.2170	1.0000							ΔWeight z-score	ρ	1.0000						
	*p*-value	0.3199									*p*-value							
Height z-score	ρ	0.3113	0.6856	1.0000						ΔHeight z-score	ρ	0.1261	1.0000					
	*p*-value	0.1816	0.0012								*p*-value	0.6069						
BMI z-score	ρ	0.5076	0.9059	0.5237	1.0000					ΔBMI z-score	ρ	0.8784	−0.4246	1.0000				
	*p*-value	0.0188	0.0000	0.0178							*p*-value	0.0000	0.0620					
Body fat	ρ	0.5412	0.6870	0.3470	0.8021	1.0000				ΔBody fat	ρ	0.7368	−0.3167	0.7761	1.0000			
	*p*-value	0.0210	0.0020	0.1872	0.0001						*p*-value	0.0012	0.2334	0.0001				
√ghrelin	ρ	−0.5180	−0.3533	−0.2886	−0.4493	−0.3435	1.0000			Δ√ghrelin	ρ	−0.1988	0.0019	−0.1848	−0.1247	1.0000		
	*p*-value	0.0080	0.0982	0.2172	0.0410	0.1642					*p*-value	0.3751	0.9963	0.4225	0.6245			
Log-Lept	ρ	0.1766	0.4707	0.2977	0.6351	0.5896	−0.1724	1.0000		ΔLog-Leptin	ρ	0.4534	0.3963	0.0364	0.5901	0.0142	1.0000	
	*p*-value	0.3984	0.0234	0.2024	0.0020	0.0101	0.4100				*p*-value	0.0341	0.0930	0.8789	0.0135	0.9475		
Log-Insulin	ρ	0.6031	0.5012	0.0023	0.6152	0.4032	−0.5134	0.4025	1.0000	ΔLog-Insulin	ρ	0.0704	0.1182	−0.0105	−0.2754	0.0725	0.0051	1.0000
	*p*-value	0.0049	0.0288	0.9931	0.0095	0.1368	0.0210	0.0792			*p*-value	0.7821	0.6751	0.9702	0.3215	0.7695	0.9852	

BMI = Body mass index. Age (years); Body fat (%); √ghrelin (pg/mL); Log-leptin (pg/mL); Log-Insulin (mU/L).

**Table 5:** Associations between nutritional status, body composition, ghrelin and leptin at the baseline and 12-months in adults.

Pre-Intervention											Post-Intervention							
		Age	Weight z-Score	Height z-Score	BMI z-Score	Body Fat	√ghrelin	Log-Leptin	Log-Insulin			ΔWeight z-Score	ΔHeight z-Score	ΔBMI z-Score	ΔBody Fat	Δ√ghrelin	ΔLog-Leptin	ΔLog-Insulin
Age	ρ	1.0000																
	*p*-value																	
Weight z-score	ρ	−0.1010	1.0000							ΔWeight z-score	ρ	1.0000						
	*p*-value	0.8990									*p*-value							
Height z-score	ρ	−0.1900	−0.7740	1.0000						ΔHeight z-score	ρ	0.7370	1.0000					
	*p*-value	0.8100	0.2260								*p*-value	0.2630						
BMI z-score	ρ	−0.0370	0.9710	−0.9010	1.0000					ΔBMI z-score	ρ	0.9654	0.5390	1.0000				
	*p*-value	0.9630	0.0290	0.0990							*p*-value	0.0350	0.4610					
Body fat	ρ	0.4510	0.7830	−0.9330	0.8670	1.0000				ΔBody fat	ρ	0.5960	0.8340	0.3940	1.0000			
	*p*-value	0.5490	0.2170	0.0670	0.1330						*p*-value	0.4040	0.1660	0.6060				
√ghrelin	ρ	0.3770	−0.9150	0.5660	−0.8500	−0.4850	1.0000			Δ√ghrelin	ρ	−0.4310	−0.9250	−0.1820	−0.8220	1.0000		
	*p*-value	0.5310	0.0850	0.4340	0.1500	0.5150					*p*-value	0.5690	0.0750	0.8180	0.1780			
Log-Lept	ρ	−0.2360	0.7650	−0.9050	0.8770	0.7110	−0.6840	1.0000		ΔLog-Leptin	ρ	0.5160	0.1090	0.6440	−0.3450	0.1090	1.0000	
	*p*-value	0.7020	0.2350	0.0950	0.1230	0.2890	0.2020				*p*-value	0.4840	0.8910	0.3560	0.6550	0.8610		
Log-Insulin	ρ	−0.891	0.3470	−0.2300	0.3630	−0.0840	−0.5910	0.6220	1.0000	ΔLog-Insulin	ρ	0.8850	0.3490	0.9621	0.3330	0.1600	0.2770	1.0000
	*p*-value	0.0420	0.6530	0.7700	0.6370	0.9160	0.2940	0.2630			*p*-value	0.1150	0.6510	0.0380	0.6670	0.7970	0.6520	

BMI = Body mass index. Age (years); Body fat (%); √ghrelin (pg/mL); Log-leptin (pg/mL); Log-Insulin (mU/L).

## Supplementary Materials

The following are available online at https://www.mdpi.com/2072-6643/11/8/1716/s1, Table S1: Time course of the diet composition.
