# Supplementary material for: Long-Term Effects of a Classic Ketogenic Diet on Ghrelin and Leptin Concentration: A 12-Month Prospective Study in a Cohort of Italian Children and Adults with GLUT1-Deficiency Syndrome and Drug Resistant Epilepsy
Source: Nutrients. 2019 Jul 25;11(8):1716. doi: 10.3390/nu11081716 (PMC6722776; doi:10.3390/nu11081716)
Supplement: Supplementary file 1 [file nutrients-11-01716-s001.pdf]

Table S1. Time course of the diet composition.

	CHILDREN AND ADOLESCENTS (n=25)							ADULTS (n=5)						
	Baseline		6 months		12 months			Baseline		6 months		12 months		
	Mean	sd	Mean	sd	Mean	sd	P-value	Mean	sd	Mean	sd	Mean	sd	P-value
Energy intake (kcal/day)	1373	518	1313	358	1335	341	0.104	1938	325	1934	313	1895	345	0.824
Energy intake/BW (kcal/kg)	61.5	21.3	58.9	23.2	59.5	22	0.874	31.6	9.5	31.7	8.6	30.8	9.8	0.792
Protein (g/day)	26.4	12.4	26.5	12.9	26.9	10.1	0.797	59.0	16.9	61,0	20.8	58.8	19.1	0.216
Protein/BW (g/kg)	1.2	0.5	1.3	0.8	1.3	0.6	0.292	1.0	0.3	1.05	0.35	1	0.3	0.894
Protein (%)	8	2	8	2	8	2	0.806	13	5	13	5	13	5	0.797
Fat (g/day)	131.3	51.0	128.2	49.0	129.4	50.5	0.090	177.6	43.5	175.8	44.1	180.2	35.3	0.806
Fat/BW (g/kg)	5.0	2.0	5.4	2.8	5.5	2.6	0.890	2.9	1.1	2.8	0.9	2.9	0.9	0.795
Fat (%)	86	5	87	6	87	6	0.798	82	8	81,0	9,0	82	8	0.810
SFA (g/day)	47.4	19.0	47.1	18.7	46.7	18.6	0.084	40.3	7.6	39.6	8.2	41	8.6	0.615
SFA/BW (g/kg)	2.0	0.7	1.6	1.1	1.9	1.2	0.196	0.6	0.2	0.8	0.4	0.7	0.4	0.579
SFA (%)	28	8	28	8	28	8	0.812	19	5	19,0	5,0	19	5	0.682
Carbohydrate (g/day)	21.6	12.6	19.8	11.2	22.5	11.8	0.474	20.1	8.7	22.8	7.9	19.8	9.2	0.189
Carbohydrate/BW (g/kg)	1.1	0.9	1.1	1	1.2	0.8	0.849	0.4	0.1	0.52	0.35	0.3	0.1	0.211
Carbohydrates (%)	7	4	7	4	7	4	0.880	4	2	4,0	3,0	4	2	0.356
Ketogenic ratio	2.9	0.8	2.7	0.7	2.7	0.5	0.500	2.5	0.9	2.3	1,0	2.4	0.6	0.192
